# Use of Indocyanine Green for Detecting the Sentinel Lymph Node in Breast Cancer Patients: From Preclinical Evaluation to Clinical Validation

**DOI:** 10.1371/journal.pone.0083927

**Published:** 2013-12-16

**Authors:** Chongwei Chi, Jinzuo Ye, Haolong Ding, De He, Wenhe Huang, Guo-Jun Zhang, Jie Tian

**Affiliations:** 1 Intelligent Medical Research Center, Institute of Automation, Chinese Academy of Sciences, Beijing, China; 2 The Breast Center, Cancer Hospital, Shantou University Medical College, Shantou, China; Glasgow University, United Kingdom

## Abstract

Assessment of the sentinel lymph node (SLN) in patients with early stage breast cancer is vital in selecting the appropriate surgical approach. However, the existing methods, including methylene blue and nuclides, possess low efficiency and effectiveness in mapping SLNs, and to a certain extent exert side effects during application. Indocyanine green (ICG), as a fluorescent dye, has been proved reliable usage in SLN detection by several other groups. In this paper, we introduce a novel surgical navigation system to detect SLN with ICG. This system contains two charge-coupled devices (CCD) to simultaneously capture real-time color and fluorescent video images through two different bands. During surgery, surgeons only need to follow the fluorescence display. In addition, the system saves data automatically during surgery enabling surgeons to find the registration point easily according to image recognition algorithms. To test our system, 5 mice and 10 rabbits were used for the preclinical setting and 22 breast cancer patients were utilized for the clinical evaluation in our experiments. The detection rate was 100% and an average of 2.7 SLNs was found in 22 patients. Our results show that the usage of our surgical navigation system with ICG to detect SLNs in breast cancer patients is technically feasible.

## Introduction

A sentinel lymph node (SLN) is the first lymph node (LN) or a group of LNs draining from the breast [1]. Since SLN mapping, as introduced for the management of breast cancer by Giuliano et al., is currently thought to be the standard for staging clinically negative axilla [2-4], sentinel lymph node biopsy (SLNB) for breast cancer is a promising surgical technique to avoid unnecessary axillary lymph node (ALN) dissection, leading to the improvement of post-operative quality of life [5]. Presently, three detection reagents are used for detecting SLNs in clinical settings: (1) blue dye, which is widely used due to its inexpensiveness, but has limited ability to visualize afferent lymphatic vessels and SLNs [6]; (2) radioactive colloids, which require a physician specializing in nuclear medicine, to use a handheld gamma counter, making SLN localization difficult; and (3) indocyanine green (ICG), which gives a fluorescent signal has been used by several other groups for locating SLNs in breast cancer to give a reliable higher signal-to-background ratio (SBR) [7-20], and in addition cheap and of low toxicity [7,21,22]. The fluorescent dyes, such as ICG, with a high SBR and detection depth in real-time observation enable superior SLN detection compared to the other two reagents. The only concern of the drug is allergic reaction occasionally [23].

Since ICG emits near-infrared (NIR) fluorescence, undetectable by human eyes, an appropriate intraoperative detection system is required to produce real-time images with a high SBR. Current systems, in breast cancer research, use a single-camera for NIR imaging , requiring the shadowless surgical lights to be turned off to detect SLNs, and then turned on to resume the surgery. This on/off switching of shadowless light is inconvenient and extends the duration of the operation. A system with three-cameras has advantages in multispectral imaging and superior background noise reduction [2,6,24-28]. However, the complexity of the system in reducing background noise increases the number of operation complication during surgery [26-29]. Therefore, an easily operated system that provides real-time images with a high SBR is needed.

In this study, we proposed a new surgical navigation system with two cameras for high SBR real-time imaging. To ensure convenience and practicality for clinical application, our surgical navigation system was designed to localize the SLN by providing an automatic real-time color and fluorescent image display recording during surgery. We assessed ICG as a fluorescent dye for SLN detection. In order to identify an optimized dose/concentration of ICG for future clinical use, we performed a preclinical test, in which serial ICG dilutions are evaluated in SLN resection experiments in nude mice and rabbits. Subsequently, clinical studies were further conducted in breast cancer patients to evaluate the feasibility of the surgical navigation system in detecting SLNs. Our study provides evidence that our surgical navigation system could provide surgeons with real-time images to accurately locate and resect SLNs during surgery. 

## Materials and Methods

### Surgical navigation system

**Figure 1:**
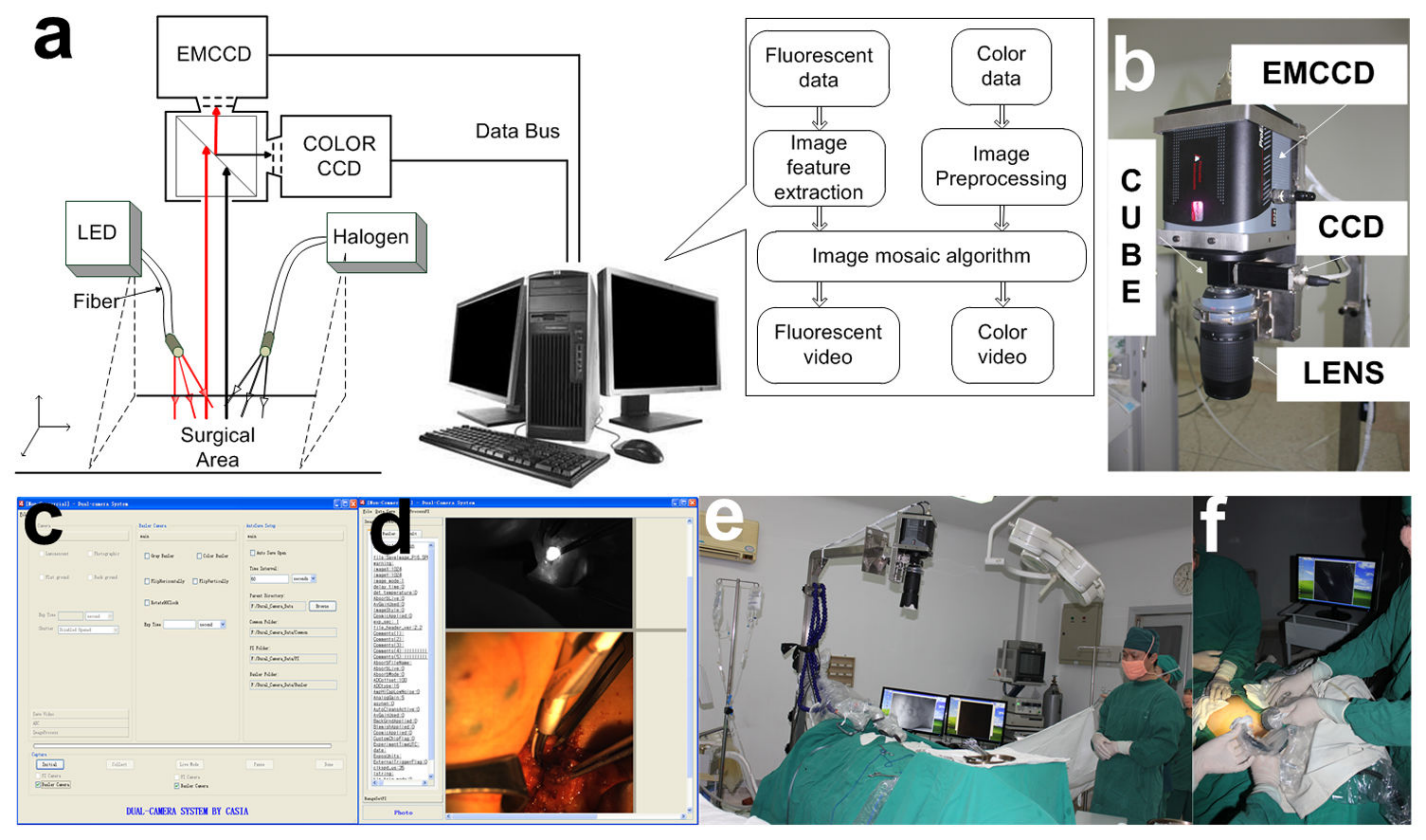
Schematic diagram of the surgical navigation system and its application in surgery. a. The principle figure of the surgical navigation system clarifying the operation course of the system. When the LED light illuminated the surgical area, the ICG dye emitted NIR light. The emission and reflection of the halogen light went through the lens to the prism. Then, the light was equally divided into two beams by the prism. One beam went through the filter to the color CCD and the other to the EMCCD. All of the data collected from the CCD were transferred to the computer, and the computer controlled the CCD. b. The hardware of the surgical navigation system. c. User interface of the software offering exposure time and auto capture interval time parameter settings. d. Image acquisition interface as an example of the capture mode results. e. Preoperative preparation in the operating room. f. Intraoperative diagnosis with a surgical navigation system carried out during the surgery.

The prototype surgical navigation system (Figure 1a) used in this study was developed by the Institute of Automation, Chinese Academy of Sciences (CASIA). The system had two charge-coupled device (CCD) cameras: an electron-multiplying CCD (EMCCD) was used to collect NIR fluorescent images (ProEM 1024B Excelon, Princeton Instrument, USA); a color CCD was used to collect visible color images (Pilot piA1400-17gc, Basler, Germany), in which two beams of light, separated by a beam splitter cube (NT49-683, Edmund Optics, USA), were received by the camera that then produced the video images on an external monitor. A fluorescence filter (wavelength 810nm-870nm) was placed in front of the EMCCD camera, while a visible light filter (wavelength 400nm-650nm) was put in front of the color CCD camera. The F-mount flange distance of the Nikon lens (Nikon Nikkor 70-300mm, f/4.5-5.6G) was 46.5mm and was chosen for this design for clear imaging. The hardware parts are shown in Figure 1b. 

Illumination was provided by a light emitting diode (LED) (NIR light source, center wavelength 760nm, maximum power 20W) for fluorochrome excitation and a 150W halogen lamp (KL1500LCD, SCHOTT, Germany) for white light color imaging. The white light was also coupled with a fiber optic bundle and homogenized by a beam expander. According to the separation of the light spectrum, it was ensured that when the shadowless surgical light was closed, the doctors could see the operative field via a halogen light. Under these conditions, the visible light did not affect the fluorescence imaging results. 

Based on the hardware system, the surgical navigation system control software was developed in two modes. One was the real-time video imaging mode, in which the color and fluorescence real-time videos were displayed separately. The other was the camera capture mode. After setting the parameters of the two cameras, one click could complete the simultaneous acquisition of the two camera images. The user interface (Figure 1c) provided complete control of the system including the camera EM mode, exposure time, image display, image overlay and image archiving. In the camera capture mode, after a set of images was acquired, the fluorescence and color images were displayed (Figure 1d). All pictures and videos could be acquired automatically during the surgery. The image processing function was designed in the software. The registration point of white light and fluorescent images was automatically calculated by the similarity matching algorithm. Then, these two images converged according to the results in the software. This feature allowed doctors to clearly locate the lesion. 

### Imaging Agents

ICG was purchased from the Yichuang Pharmaceutical Limited Liability Company (Dandong, China). To prevent exposure to the sunlight and fluorescence bleaching, the ICG was stored at 4°C. Two hours prior to surgery, 25 mg ICG was dissolved in 5 ml of water to yield a concentration of 5 mg/ml, which was an optimal concentration based on our preclinical study.

### Lymphatic mapping in animal models

 The Institutional Animal Care and Use Committee of Shantou University Medical College approved all animal studies. Five nude mice (nu/nu, Vital River Laboratory Animal Technology Co., Ltd., Beijing, China) and 10 rabbits (New Zealand white rabbits, Experimental Animal Center of Guangdong, Foshan, China) were used to map the lymphatic vessels and lymph nodes. In the mouse study, female mice were anesthetized by an injection of a 0.2mL mixture of ketamine, xylene and sterile distilled water at a ratio of 7:3:4. Fluorescent images were acquired with the imaging system after subcutaneous injection of 0.1mL ICG on the right side of the second mammary pad. After gently massaging the surrounding breast tissue to develop the lymph channels the skin was cut and the SLNs were removed.

Similarly, female New Zealand rabbits were anesthetized with an injection of sodium pentobarbital (30 mg/kg intravenously) through the ear vein and placed in a prone position on a fixation bed. After the rabbits were sedated (typically 4–5 min), fluorescent images were then acquired after subcutaneous injection of 0.1 ml ICG around the areola. A varied concentration of ICG was administered into the second armpit areola to locate the SLN and to quantitate the light intensity. The light intensity statistics were performed using the Prism 5.0 (GraphPad-Prism) computer program.

### Patient characteristics and surgical procedure

Twenty-two breast cancer patients, ranging from 32 to 68 years of age (median age of 49 years) with early stage breast cancer were admitted to the Breast Center of the Cancer Hospital of Shantou University Medical College and were enrolled in the study. Of those, twelve (54.5%) were premenopausal women and ten were postmenopausal (45.5%). All patients had a tumor size less than 5 cm (i.e., T1-2N0M0) and negative lymph nodes, and were eligible for sentinel lymph node biopsy (SLNB). Informed consent was given formally to all patients before surgery. All subjects gave written informed consent after the experimental procedures were fully explained. This study was approved by the Institutional Review Board (IRB) of the Cancer Hospital of Shantou University Medical College and performed in accordance with the ethical standards of the Declaration of Helsinki. 

Before surgery, the surgical navigation system was moved above the operating field and prepared for imaging where the NIR light source and halogen fiber were covered by sterile sheets. In this setting, the lens of the camera was situated approximately 60 cm above the patient. Preoperative and intraoperative pictures are shown in Figures 1e and f. The injection concentration of ICG was 5mg/ml, which was based on a published paper and our pre-clinical trials [30]. ICG was subcutaneously injected into 2-4 points of the areola. With continuous massage for 5-10 minutes, the lymph vessels connecting to the injection point were visualized, using the surgical navigation system, along with a light spot showing SLN on the fluorescent image window. With the assistance of the video showing the maximum gray value, the surgeons could trace the SLN according to the fluorescent image. SLNs detected were then removed under the navigation of real-time NIR fluorescence imaging. The excised SLNs were also examined by NIR imaging, and then sent in for pathological examination.

## Results

### Surgical navigation system test

The spatial resolution, field of view and working distance were the main parameters tested after finishing the prototype of the surgical navigation system. The detailed specifications of the system are shown in Table 1. In a practice setting, the distance between the LED light source and surgical area was around 10-20cm; if the distance was beyond 20cm, the fluorescent image became indistinguishable. The results of the standard television card tests with this system are shown in Figure S1.

**Table 1:** Specifications of the surgical navigation system.

System Features	Performance parameters
Chip area	EMCCD 1.3’’; COLOR CCD 2/3’’
Lens focal length	70-300mm
Working distance	600mm
Surgical field of view	79mmW*79mmH to 125mmW*125mmH
Sensor resolution (spatial)	1024*1024 imaging pixels
	13 x 13 μm pixels
	13.3 x 13.3 mm imaging area (optically centered)
Sensor resolution (temporal)	8.5fps(full frame),16.7fps(binning 2Xver)
Image windows	Visible, fluorescent, overlay

### Optimized dose/concentration of ICG *in vivo*

**Figure 2:**
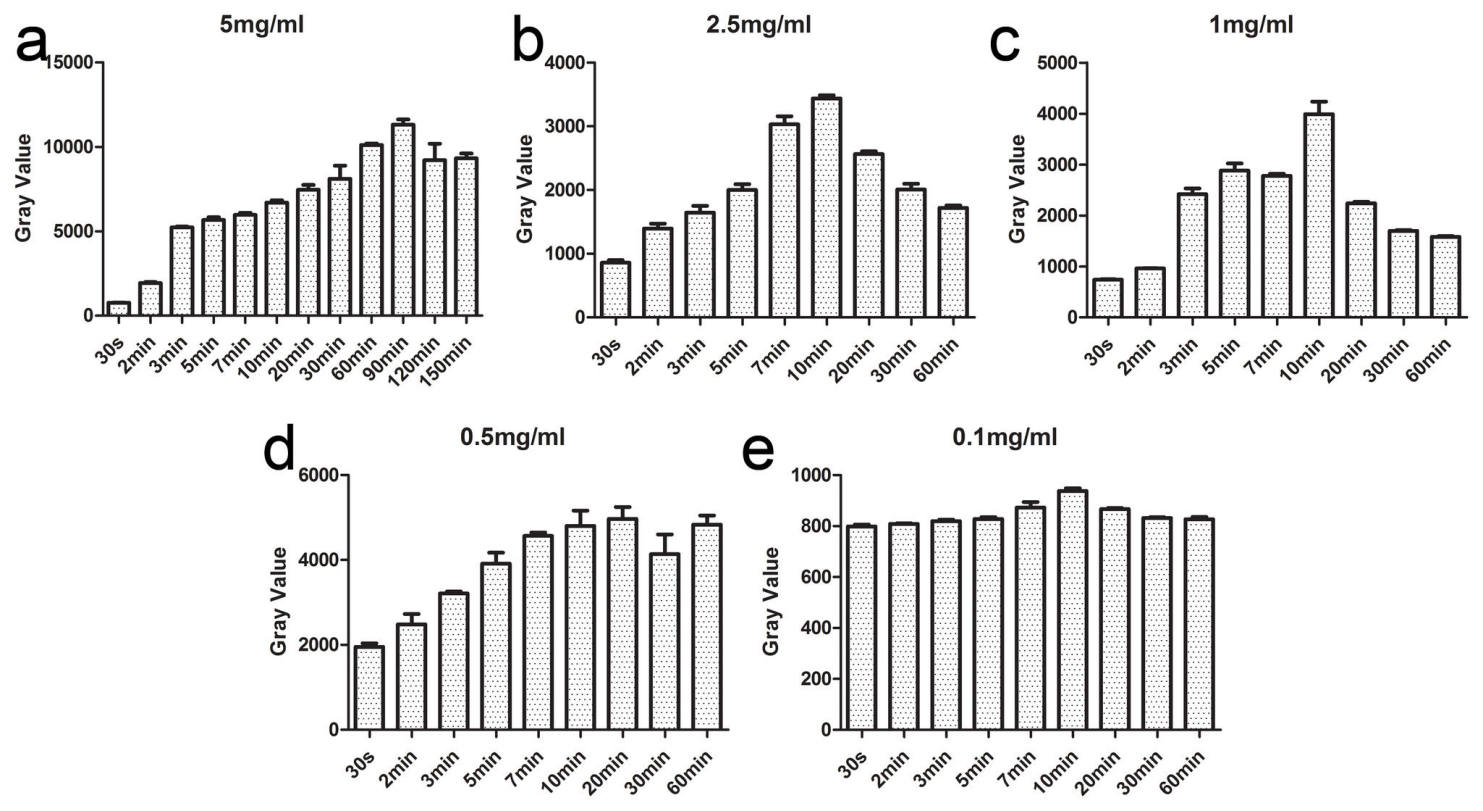
Pharmacokinetic experiments on rabbits. Five pharmacokinetic experiments using ICG with five concentrations were done on rabbits with the injection doses of 0.1 ml in the areolar area. Our software to evaluate the changes of the light intensity value at each time point tested the light intensity of SLN in rabbits. The results are shown in Figures a-e. The error bars mean the variance of light intensity from 3 rabbit experiments per group at a certain concentration and time point. Then, we entered data into the computer program Prism 5.0 (GraphPad-Prism). The light intensity statistics were performed using the software. From the results we could obtain the time point of the surgery and the effective time of the operation.

SLNs could be detected as early as 3 to 5 minutes after injection and the enabling time for surgery was 10 minutes after injection (Figures 2a to e). The peak of intensity at a concentration of 5mg/ml and dose of 0.1ml of the ICG solution appeared 90 minutes after injection (Figure 2a). Based on our experiments, we chose three concentrations (0.025mg/ml, 1mg/ml, and 5mg/ml) for a feasibility test. In preclinical studies, strong light intensity was visible in the EP tube at a concentration of 0.025mg/ml *in vitro*, but could not be detected *in vivo*. The 1mg/ml concentration was suitable for animal detection, but sufficient for clinical usage. Finally a concentration of 5mg/ml was recommended for clinical use due to its strong light intensity and long duration.

### Detection of SLN by ICG in nude mice *in vivo*

**Figure 3:**
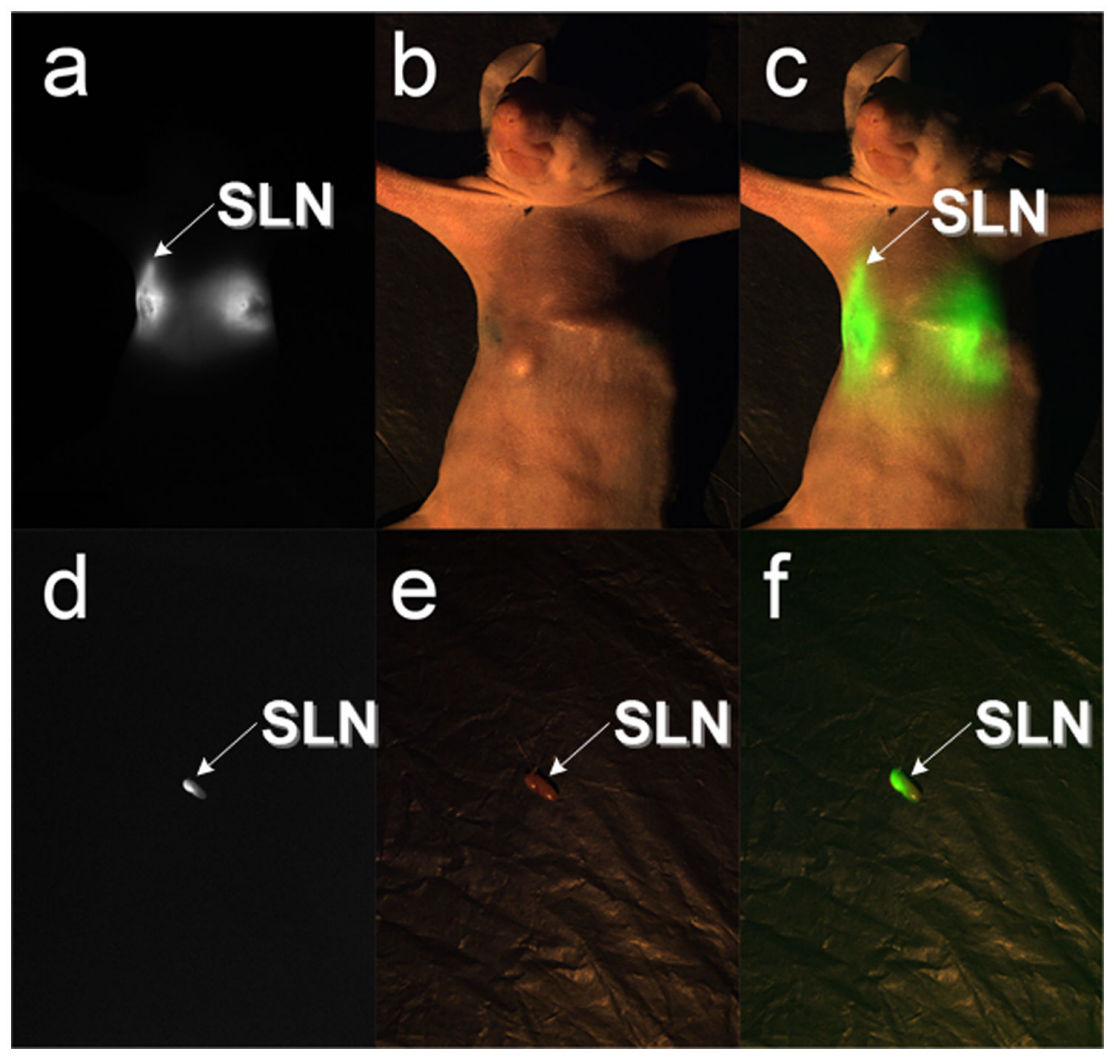
The SLN resection experiments in nude mice. There were two groups of figures directly acquired by the CCD cameras. The last column images were obtained after the processing of the two images in the front. When the ICG solution with a concentration of 1mg/ml was injected into the third areola of a nude mouse, ten minutes later we got a. fluorescent image for mapping the SLN. At the same time, we got b. the color image. According to the software computation, the pseudo-green fluorescent image was overlaid on top of the color image. The result of the image fusion was c. the overlay image. After dissection of the SLN, d. fluorescent image and e. color image were acquired simultaneously. From the f. overlay image, we could clearly see the fluorescence information in the color image. All dissections were sent in for pathological examination. All of the tissue sections were judged to be the SLN.

To determine the feasibility of the system in a preclinical setting, 0.1mL of a 1mg/ml ICG solution was injected into the armpit of a mouse (Figure 3). The fluorescent and visible images are shown in Figures 3a and b. Using the software alignment operation, the fluorescent image was overlaid with the visible image (Figures 3c and f). Finally, the light-emitting tissue was confirmed as the SLN (Figures 3d and e) by pathological examination.

### *In vivo* detection of SLN by ICG in rabbits

**Figure 4:**
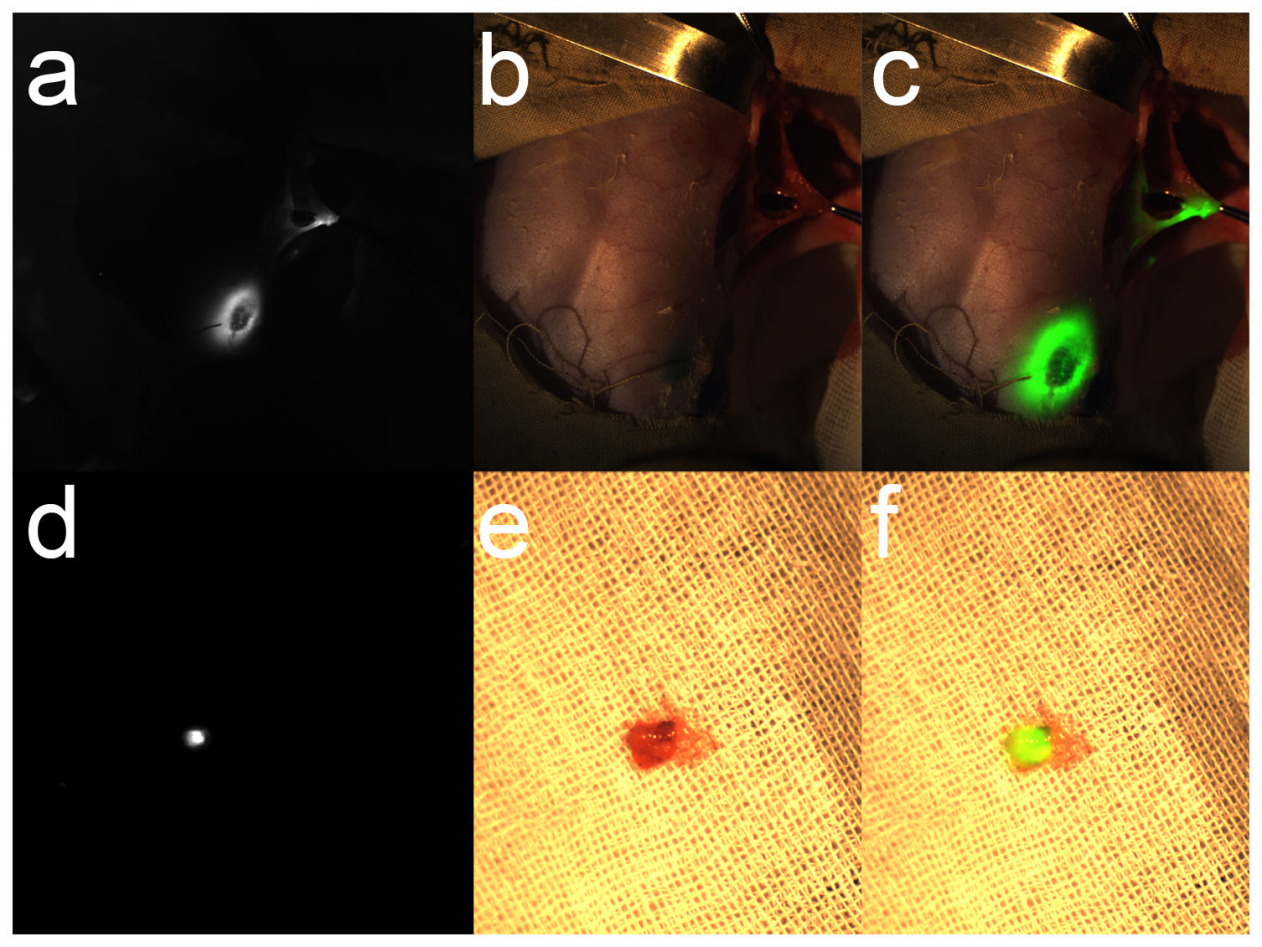
The SLN resection experiments in rabbits. When the SLN was dissected, we took a photo as shown in a. fluorescent image. The b. color image was acquired at the same time. c. The overlay image also showed the fluorescent position in the color image. After resection, the SLN was put on medical gauze. It was shining brightly as d. the fluorescent image. Although in the e. color image there was no difference in the light, the f. overlay image showed that it was illuminated. All of the dissections were sent in for pathological examination. All of the tissue sections were judged to be the SLN.

Five experiments were conducted on rabbits to ensure that SLNs could be located by using ICG. During the surgical experiment, real-time videos were shown on the monitor (Figures 4a and b). Figure 4c is the overlay of the pseudocolor fluorescent signal on top of the color image. Following excision of the SLN, a fluorescent image was taken (Figure 4d). The visible image and the merged image are shown in Figures 4e and f. The video of the fluorescence for the entire surgical procedure is shown in Video S1. 

### Detection of SLN in breast cancer patients by ICG

**Figure 5:**
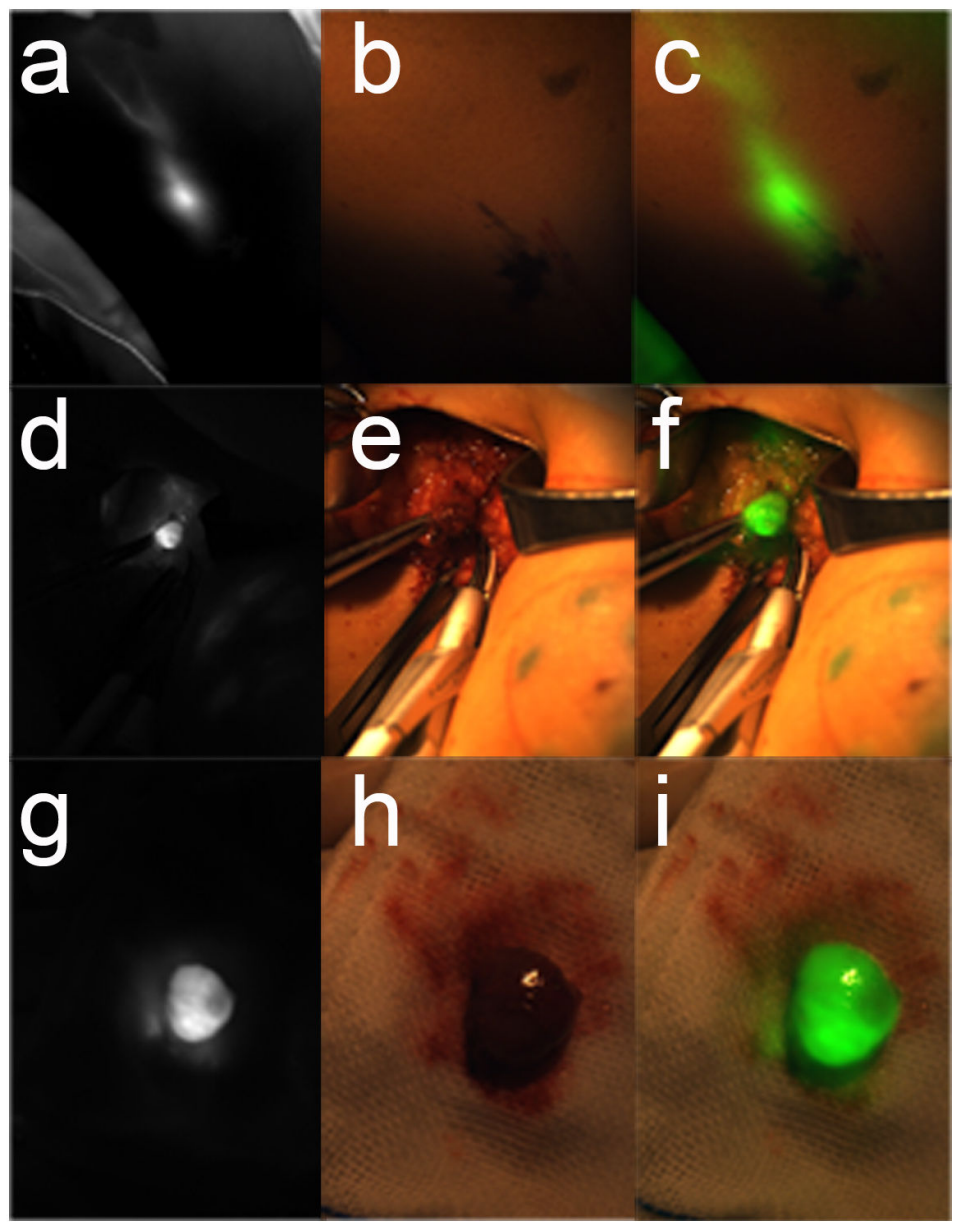
ICG-guided intraoperative detection and resection of the SLN in humans. According to the preclinical trials, 22 cases of patients were taken from the SLNB surgery. In the beginning, the ICG solution was injected into the areolar region. About 3 minutes later, the lymphatic drainage and SLN would be clearly displayed on the monitor as shown in a. the fluorescent image *in* *vivo*. Because near-infrared light is not visible, there was no light information in the b. color image *in* *vivo*. Through the software of the surgical navigation system, the location of the SLN is shown in c. where the overlay image *in* *vivo* could be distinguished accurately. According to the guidelines of the fluorescent image, the surgery could quickly find the location of the SLN. d. The fluorescent image was captured before dissection. From the e. color image and the f. overlay image, SLN could be located with tweezers. The SLN was carefully removed and put on gauze. With the near-infrared light irradiation, the SLN was bright as shown in g. the fluorescent image during dissection. Such a visible image was displayed in h. the color image during dissection. Finally, the merged image of the pseudo-green fluorescence image and the color image is shown in i. the overlay image during dissection. All dissections were sent in for pathological examination. All of the tissue sections were judged to be the SLN.

Clinical statistical results are shown in Table 2. Five minutes after injection, the draining lymph vessels and SLNs were visualized on the fluorescent image window, using the color and overlay images (Figure 5). According to the light-emitting position, an incision was executed with a scalpel. Then the light-emitting region could be targeted with the assistance of our system. The surgeons were able to identify the SLN according to the fluorescent image (Figure 5d) and accurately locate it on the color image (Figure 5e). The fluorescent and color images were overlaid together by using our software (Figures 5f and i). After removal of the light-emitting region (Figure 5h), the SLN kept lighting up (Figure 5g). The surgery video is shown in Video S2. 

**Table 2:** Characteristics of 22 patients who underwent ICG-guided SLNB.

Study no.	Age	Menstrual state	Tumor location	Tumor size (cm)	cTNM	pTNM	ICG dose (ml)	SLN no.	SLN metastasis	ALN no.	ALN metastasis	Surgery	
1	43	pre	Right upper outer	3×1.9	T2N1M0	T2N1M0	2	2	1/2	11	1/11	MRM +SLNB	
2	46	pre	Left lower inner	1.2×0.8	T1N0M0	T1N0M0	1	1	0/1	32	0/32	BCS +ALND+SLNB	
3	50	pre	Left upper outer	0.5×0.3	T1N0M0	T1N0M0	1	3	0/3	21	0/21	MRM +SLNB	
4	68	post	Left upper outer	2.9×2.4	T2N1M0	T2N0M0	0.5	3	0/3	21	0/21	MRM +SLNB	
5	38	pre	Left lower outer	1.8×0.6	T1N0M0	T1N0M0	1	1	0/1	18	0/18	MRM +SLNB
6	48	pre	Right upper outer	4.0×2.8	T2N1M0	T2N0M0	1	6	0/6	30	0/30	MRM +SLNB	
7	66	post	Left upper inner	4.2×3.7	T2N1M0	T1N0M0	1	2	0/2	16	0/16	MRM +SLNB	
8	47	pre	Left lower outer	5.7×1.6	T3N1M0	T3N2M0	1	3	1/3	25	4/25	MRM +SLNB	
9	62	post	Left lower borderline	0.9×0.8	T1N0M0	T1N0M0	1	3	0/3	13	0/13	MRM +SLNB	
10	55	post	Right upper inner	4.0×4.0	T2N1M0	T2N0M0	1	4	0/4	9	0/9	MRM +SLNB	
11	59	post	Left upper inner	3.0×2.2	T2N1M0	T2N3M0	1	1	1/1	17	11/17	MRM +SLNB	
12	67	post	Right upper outer	1.8×1.6	T1N0M0	T1N1M0	1	1	0/1	12	2/12	BCS +ALND+SLNB	
13	32	pre	Right upper borderline	3.6×2.9	T2N1M0	T2N0M0	1	4	0/4	12	0/12	MRM +SLNB	
14	40	pre	Left middle	1.8×1.2	T1N1M0	T2N0M0	2	4	0/4	10	0/10	MRM +SLNB	
15	45	pre	Left lower outer	1.5×0.8	T1N0M0	T1N0M0	2	4	0/4	15	0/15	BCS +ALND+SLNB	
16	57	post	Left lower outer	2.0×1.0	T2N0M0	T1N0M0	2	3	0/3	11	0/11	MRM +SLNB	
17	33	pre	Right lower	2.3×1.2	T2N1M0	T2N1M0	2	4	1/4	20	0/20	MRM +SLNB	
18	63	post	Left upper outer	2.4×2.6	T2N1M0	T2N0M0	2	3	0/3	5	0/5	MRM +SLNB	
19	53	post	Right upper outer	2.5×1.7	T2N1M0	T2N1M0	2	2	1/2	14	0/14	MRM +SLNB	
20	33	pre	Left upper outer	3.1×1.6	T2N0M0	T2N1M0	2	1	1/1	6	0/6	MRM +SLNB	
21	50	post	Left upper	5.3×4.5	T3N1M0	T3N3M0	2	2	2/2	19	9/19	MRM +SLNB	
22	43	pre	Left upper outer	1.6×1.2	T1N0M0	T1N0M0	2	2	0/2	24	0/24	MRM +SLNB	

ALN axillary lymph node, ALND axillary lymph node dissection, SLNB sentinel lymph node biopsy, MRM modified radical mastectomy, BCS breast conserving surgery

SLNB using the surgical navigation system was performed on all patients and all resected LNs were evaluated by pathological examination. One or more SLNs were found in all 22 patients (100%). The total number of identified SLNs was 59, or 2.7 per patient (range 1-6). All 59 SLNs detected by the surgical navigation system gave a fluorescent signal and the pathology results confirmed they were LN tissues (detection rate=100%). All patients then received axillary dissection with a total of 361 LNs removed. The mean number of LNs removed was 16.4 per patient (range 9-32).

**Figure 6:**
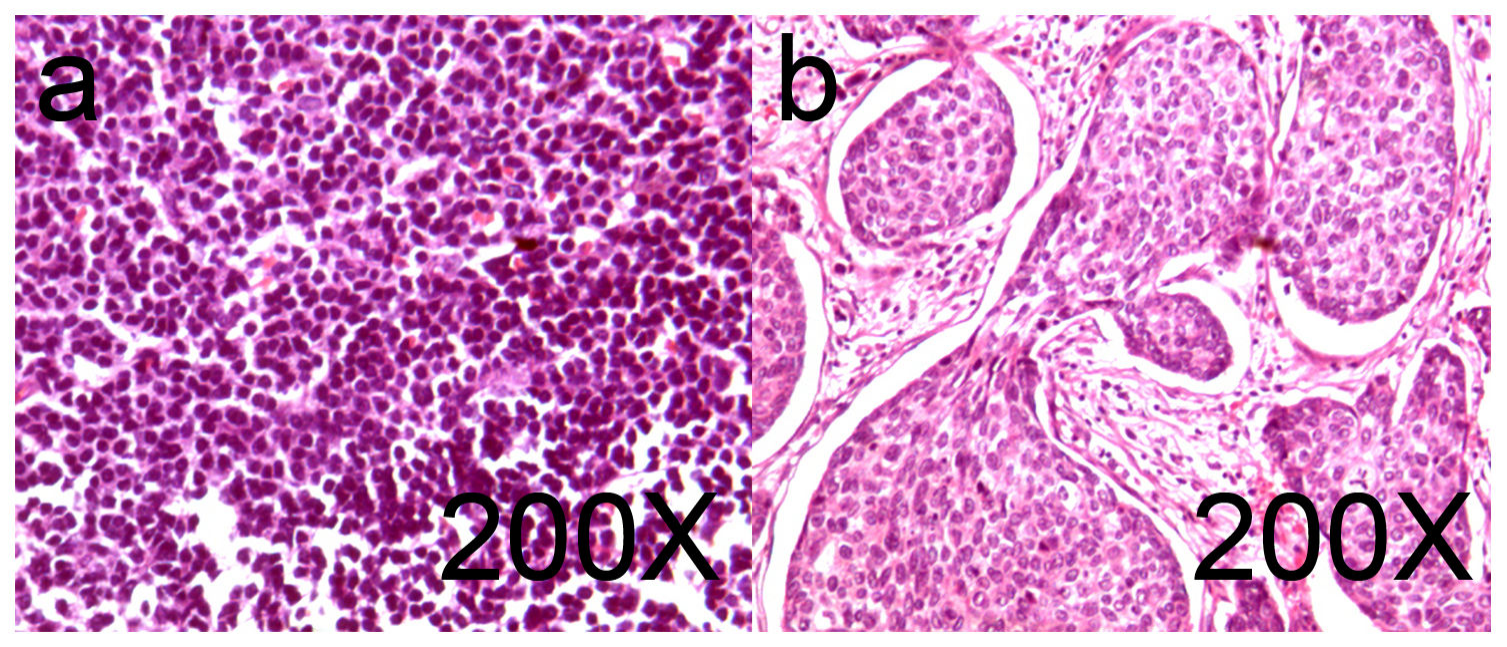
The normal and tumor-metastasis pathology slices of SLN dissected by ICG-guided surgery. All of the dissected SLNs were sent in for pathological examination. After the conventional Hematoxylin-Eosin (HE) staining, the results proved that all of the dissected tissue specimens were lymph nodes. Figure a. shows normal sentinel lymph node cells with no cancer metastasis. Figure b. shows infiltrating ductal breast cancer.

Eight out of 59 SLNs (13.6%) contained metastases, while 27 out of 361 LNs (7.5%) showed metastases. One patient possessed to have pathological LN metastases, but not SLN metastases, whereas pathological examination of another three patients revealed metastases, but not in the ALN. The slice in Figure 6a is from the patients who did not have SLN metastases. The slice in Figure 6b is from a patient whose SLN was diagnosed with metastases. No side effects were reported after the injection of ICG.

## Discussion

Radiology approaches such as X-rays, computed tomography (CT), magnetic resonance imaging (MRI), positron emission tomography (PET) and single photon emission computed tomography (SPECT) have been considered to assist surgical procedures, but most of them were non-applicable for intraoperative surgery. In contrast, the fluorescence imaging approach offers superior application of non-radiation and high resolution and sensitivity, compared with radiological imaging visual inspection and palpation during surgery [31]. So, for the intraoperative application, the instrument with fluorescence imaging would be provided with the following standards: high SBR real-time imaging of the surgical procedure, easy operation, non-radiation and non-physical contact to patients. 

Our surgical navigation system fulfilled the above criteria. Compared to other current intraoperative systems, ours not only kept the advantage of easy operation in a single-camera system with improved quality of imaging and convenience of operation during surgery, but also ensured the core function of high SBR real-time imaging in three-camera systems. In comparison of new approach named goggle system [32], our system takes the advantage of high image resolution and low temporal noise. Therefore, with the guidance of our system, the surgeons could accurately and rapidly locate the SLN with the high quality visible images and the fluorescent images during the surgical procedure. 

Since the detection rate was 100% and an average of 2.7 SLNs was detected in all patients in our study, this demonstrated that SLN detection with our system was practical and applicable, especially for the early stage breast cancer patients. It was reported that, a combination of radioactive colloid and blue dye was used for SLN mapping, which ensured the identification rate reaching 95%-97%, while each detection method had certain obvious shortcomings resulting in significantly lower identification rates by themselves [8,10,33-38]. With the usage of ICG in our study, the detection rate was 100%, which was almost the same as or even better than using the combination method. In future work, we plan a large sample study using our system to specifically compare ICG and blue dyes in terms of testifying their sensitivity and specificity during the surgical procedure [8].

To explore the optimized injection dosage and time of ICG for system detection, a series of trials was performed in our preclinical study. The results showed that, the SLN could be clearly visualized by using 1mg/ml ICG solution. However, the light intensity and duration were not enough for surgical purposes. Pre-clinical trials showed that the 5mg/ml ICG solution had strong fluorescence light intensity and the duration was long enough for 2.5 hours. The surgery of SLN dissection usually took 20-30 minutes, so that the fluorescence of ICG at 5mg/ml could be adequate for the whole process. Therefore, for clinical studies, we chose the concentration of 5mg/ml ICG solution. In future research, we will develop a surgical navigation system with improved sensitivity, which can detect weaker fluorescent signal and perform experiments that clarify the lowest concentration of the solution suitable for clinical use.

Although our surgical navigation system proved feasible in detecting SLN in breast cancer research with the usage of ICG, it also took the potential of application in other clinical areas, such as cervical cancer SLN detection studies [39]. 

In the future, with the aid of our surgical navigation system, we will try to dissect the orthotopic breast tumor by using the targeted NIR probe, which can distinguish the margins between the tumor and normal tissues, and guide the surgical resection appropriately [40,41]. 

## Conclusions

In this paper, to solve the problem of positioning the SLN in early stage breast cancer research, we have developed a surgical navigation system and presented an efficient method for detecting SLN during surgeries with the advantage of real-time tracing of lymph flow and the one-step procedure. Accurate navigation and reliable treatment results will aid surgeons with better judgment during surgery. Our approach delivers valuable information and provides a useful method that facilitates more detailed exploration for surgical navigation research.

## Supporting Information

Figure S1 **The resolution test of the surgical navigation system.** The aim was to find the highest spatial frequency at which two lines could be distinguished from each other. Figure a (color image) and b (fluorescent image) were the images taken by the surgical navigation system. Figure c whose horizontal axis represents the pixel number and the vertical axis represents the gray value was the analysis of Figure a. Correspondingly, Figure d showed the analysis results of Figure b. The numbers, such as 350, 400, 550…, in figure a and b were the television line numbers which represented the ability of the resolution of the video system.
(TIF)

Video S1 **This is a video of ICG-guided removal of the SLN in the rabbit.** It includes the whole course of finding the SLN in the rabbit. This was the epitome of all rabbit removal experiments.
(MOV)

Video S2 **This is a video of ICG-guided surgery of the SLN in humans.** The course was just like the experiment for the rabbit. It also includes the whole course of finding the SLN in humans during surgery. Lymphatic vessels and SLN are clearly displayed in this video.
(MOV)
