# Supplementary figures and images for: Use of Indocyanine Green for Detecting the Sentinel Lymph Node in Breast Cancer Patients: From Preclinical Evaluation to Clinical Validation
Source: PLoS One. 2013 Dec 16;8(12):e83927. doi: 10.1371/journal.pone.0083927 (PMC3865279; doi:10.1371/journal.pone.0083927)

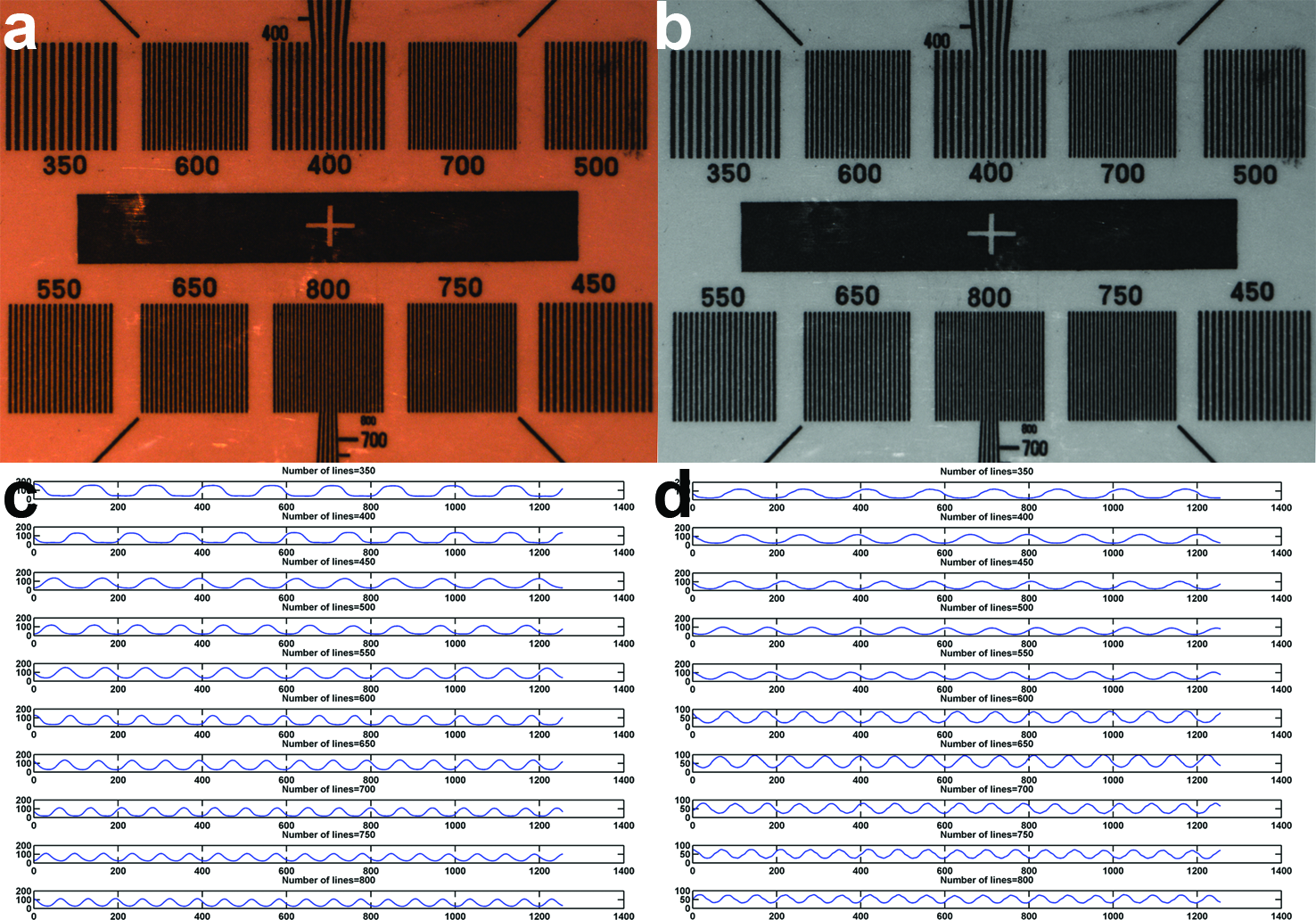

Supplement: Figure S1 — The resolution test of the surgical navigation system. The aim was to find the highest spatial frequency at which two lines could be distinguished from each other. Figure a (color image) and b (fluorescent image) were the images taken by the surgical navigation system. Figure c whose horizontal axis represents the pixel number and the vertical axis represents the gray value was the analysis of Figure a. Correspondingly, Figure d showed the analysis results of Figure b. The numbers, such as 350, 400, 550…, in figure a and b were the television line numbers which represented the ability of the resolution of the video system. (TIF) [file pone.0083927.s001.tif]
